# Impact of Admission Blood Glucose on Coronary Collateral Flow in Patients with ST-Elevation Myocardial Infarction

**DOI:** 10.1155/2018/4059542

**Published:** 2018-03-12

**Authors:** Ozge Kurmus, Turgay Aslan, Berkay Ekici, Sezen Baglan Uzunget, Sukru Karaarslan, Asli Tanindi, Aycan Fahri Erkan, Ebru Akgul Ercan, Celal Kervancıoglu

**Affiliations:** Department of Cardiology, Ufuk University Faculty of Medicine, Ankara, Turkey

## Abstract

In patients with acute myocardial infarction, glucose metabolism is altered and acute hyperglycemia on admission is common regardless of diabetes status. The development of coronary collateral is heterogeneous among individuals with coronary artery disease. In this study, we aimed to investigate whether glucose value on admission is associated with collateral flow in ST-elevation myocardial infarction (STEMI) patients. We retrospectively evaluated 190 consecutive patients with a diagnosis of first STEMI within 12 hours of onset of chest pain. Coronary collateral development was graded according to Rentrop classification. Rentrop 0-1 was graded as poor collateral development, and Rentrop 2-3 was graded as good collateral development. Admission glucose was measured and compared between two groups. Mean admission glucose level was 173.0 ± 80.1 mg/dl in study population. Forty-five (23.7%) patients had good collateral development, and 145 (76.3%) patients had poor collateral development. There were no statistically significant differences in demographic characteristics between two groups. Three-vessel disease was more common in patients with good collateral development (*p*=0.026). Mean admission glucose level was higher in patients with poor collateral than good collateral (180.6 ± 84.9 mg/dl versus 148.7 ± 56.6 mg/dl, resp., *p*=0.008). In univariate analysis, higher admission glucose was associated with poor collateral development, but multivariate logistic regression analysis revealed a borderline result (odds ratio 0.994, 95% CI 0.989–1.000, *p*=0.049). Our results suggest that elevated glucose on admission may have a role in the attenuation of coronary collateral blood flow in acute myocardial infarction. Further studies are needed to validate our results.

## 1. Introduction

In patients with acute myocardial infarction (MI), glucose metabolism is altered and acute hyperglycemia on admission is common regardless of diabetes status [1, 2]. Among patients without a history of diabetes, admission hyperglycemia (AH) may be resulting from previously undiagnosed diabetes mellitus or glucose intolerance, stress response mediated by cortisol and noradrenaline, or combination of these [3, 4]. Several studies have demonstrated that AH is independently associated with increased mortality after MI, regardless of treatment modality [5–7]. It has also been reported that nondiabetic patients with MI and AH have higher rates of heart failure, cardiogenic shock, and ventricular tachycardia [8–11].

Formation of coronary collateral vessels is an adaptive response secondary to myocardial ischemia in the presence of significant stenosis or total occlusion. It can provide an alternative source of blood supply to a myocardial area jeopardized by ischemia. It has been reported that poor collateral development is associated with larger infarct size, increased mortality, higher rates of ventricular aneurysm, and arrhythmia [12–16]. Poor collateral development despite significant stenosis suggests that multiple factors affect collateral circulation besides coronary artery disease severity.

The aim of this study was to evaluate the association between admission glucose and coronary collateral development in patients with ST-elevation myocardial infarction (STEMI).

## 2. Methods

This is a retrospective study. It was approved by the local ethics committee. Between January 2016 and January 2017, 190 patients with a diagnosis of first STEMI who were admitted to our institution were enrolled in this study. STEMI was diagnosed as typical chest pain lasting 30 minutes or more and ST segment elevation of 1 mm or more in at least two contiguous leads or 2 mm or more in leads V1 through V3 on the electrocardiography (ECG). Patients with complete LBBB on admission ECG were excluded. Demographic and clinical data including age, gender, prevalence of DM, hypertension, dyslipidemia, smoking history, and type of myocardial infarction were obtained from all patients. All patients underwent coronary angiography within 12 hours of onset of chest pain for primary percutaneous coronary intervention and received recommended standard management for STEMI. The admission plasma glucose concentrations from blood samples drawn on arrival in the emergency room were recorded. Exclusion criteria were as follows: STEMI > 12 hours from onset of chest pain, previous coronary revascularization history, history of malignancy or inflammatory disease, evidence of infectious disease, severe hepatic or renal insufficiency, and receiving steroids. None of the patients were receiving long-acting nitrates or intravenous nitrate infusion before angiography. Also, patients with incomplete data or without available glucose at admission were excluded.

### 2.1. Definitions

The assessed clinical parameters were age, gender, and coronary risk factors. Hypertension was defined as systolic blood pressure ≥140 mmHg and/or diastolic blood pressure ≥90 mmHg and/or current medication with antihypertensive drugs. Patients were defined as diabetic if they had been informed of this diagnosis before and had been using oral antidiabetic drugs or insulin treatment on admission. Hyperlipidemia was defined as serum total cholesterol > 200 mg/dl or serum triglyceride > 150 mg/dl or previously diagnosed and treated hypercholesterolemia. Body mass index (BMI) was calculated as body weight in kilograms divided by the squared value of body height in meters (kg/m^2^).

### 2.2. Angiographic Procedure and Coronary Collateral Grading

Coronary angiography was performed via the femoral artery for all patients using the Judkins technique within 12 hours of the onset of the chest pain. Infarct related artery (IRA), coronary stenosis, and collateral grading were estimated by two independent cardiologists who were blinded to the clinical information and laboratory parameters of the patients. IRA was totally occluded in all patients. Patients with single-vessel disease had only occlusion in IRA. Patients with two- and three-vessel diseases had occlusion in IRA and ≥70% stenosis in other epicardial vessels. Coronary collateral development was graded according to Rentrop classification: 0 = no filling of any collateral vessel; 1 = filling of the side branches of the artery to be perfused by collateral vessels without visualization of the epicardial segment; 2 = partial filling of the distal epicardial segment by collateral vessels; and 3 = complete filling of the distal epicardial segment by collateral vessels. Rentrop 0-1 was graded as poor collateral development, and Rentrop 2-3 was graded as good collateral development [17].

### 2.3. Statistical Analysis

Continuous variables were expressed as mean ± SD; categorical variables were defined as numbers and percentages. Student's *t*-test was used to compare continuous variables. Differences in the distribution of categorical variables were assessed by chi-square analysis. First, univariate analysis was performed. Subsequently, multivariate regression analysis was performed to determine independent predictors of coronary collateral development. Variables with a *p* value of <0.25 on univariate analysis were included in multivariate logistic regression analysis. A *p* value of <0.05 was considered statistically significant.

## 3. Results

The mean admission glucose level was 173.0 ± 80.1 mg/dl in all study population. The mean admission glucose level was 148.3 ± 52.0 mg/dl in nondiabetic patients and 238.6 ± 102.4 mg/dl in diabetic patients (*p* < 0.001). Forty-five (23.7%) patients had good collateral development and 145 (76.3%) patients had poor collateral development. The two groups did not differ for age, male gender, diabetes mellitus (DM), hyperlipidemia, hypertension, body mass index, smoking history, and chest pain to angiography time. The demographic and laboratory characteristics of the groups are shown in Table 1. Three-vessel disease, nonanterior myocardial infarction, and preinfarction angina were more common in patients with good collateral development (*p*=0.026, *p*=0.036, and  *p*=0.041, resp.). The mean low-density lipoprotein level was higher in poor collateral group than in good collateral group. There was no difference between two groups in terms of ejection fraction (*p*=0.476). The mean admission glucose level was higher in patients with poor collateral than in good collateral (180.6 ± 84.9 mg/dl versus 148.7 ± 56.6 mg/dl, resp., *p*=0.008). In univariate analysis, higher admission glucose was associated with poor collateral development, but multivariate logistic regression analysis revealed a borderline result (odds ratio 0.994, 95% CI 0.989–1.000, *p*=0.049). Multivessel disease and preinfarction angina were associated with good collateral development in multivariate analysis (odds ratio 0.910, 95% CI 0.754–0.989, *p*=0.037 and odds ratio 0.932, 95% CI 0.816–0.985, *p*=0.043) (Table 2).

## 4. Discussion

In our study, we investigated whether admission glucose is related to collateral development in STEMI patients. We found that the admission glucose was higher in patients with poor collateral vessels than with good collateral vessels.

Regardless of diabetes status, hyperglycemia is a common finding during the admission of patients with acute MI. The increased release of stress hormones during the first hours of acute MI leads to inhibition of insulin secretion and increased insulin resistance, thus inducing stress hyperglycemia [4]. However, among patients with no history of DM, hyperglycemia at admission may reflect previously undiagnosed DM or impaired glucose tolerance [3].

It has been reported that AH is associated with an increased risk of in-hospital mortality and worse long-term prognosis in patients with acute MI [18–20]. It has also been reported that AH is associated with increased incidence of heart failure, larger infarct size, and impaired predischarge left ventricle ejection fraction in acute MI patients [8]. But, the mechanisms underlying these associations are not fully understood, and whether hyperglycemia is causally related to adverse events after acute MI is unclear.

A well-developed coronary collateral limits ischemia, reduces the size of myocardial infarction, preserves left ventricle function, and has a favourable impact on the prognosis of patients with coronary artery disease [13, 21]. But, there is notable variation in the degree of coronary collateral development between patients with coronary artery disease. Whether acute hyperglycemia affects myocardial tissue and so mortality in acute MI patients via the collateral development remains a matter of debate.

The influence of DM on coronary collateral development has been studied, and the results of these studies have been conflicting. Some studies suggested that coronary collateral development was reduced in patients with DM [22, 23]; however, such a difference was not reported by others [24, 25].

Limited data are available on the effect of acute hyperglycemia on coronary collateral development. In an animal study, to produce acute hyperglycemia, dogs received intravenous 15% dextrose in water to increase blood glucose concentrations to 200, 400, and 600 mg/dl. Retrograde collateral blood flow was measured at each glucose level. Mild hyperglycemia (200 mg/dl) alone had no effect on retrograde flow, but moderate (400 mg/dl) and profound (600 mg/dl) hyperglycemia produced reductions in collateral flow and addition of L-arginine prevented decreases in collateral flow during hyperglycemia [26]. It was suggested that hyperglycemia impairs the nitric oxide- (NO-) mediated regulation of coronary collateral flow. In another study, it was found that the presence of blood glucose level > 350 mg/dl for 21 days abolishes development of coronary collateral vessels in dogs [27].

The exact duration and severity of hyperglycemia that inhibits collateral formation and the underlying precise mechanism is not clear. NO is essential for collateral formation [28]. In a study by Fujita et al., administering glyceryl trinitrate to the dogs with newly developed collaterals increased collateral blood flow during a transient period of coronary occlusion [29]. It was shown that hyperglycemia, whether acute or chronic, impairs NO availability and endothelium-dependent vasodilation and enhances the production of endothelial-derived vasoconstrictor prostanoids [30, 31]. Also, experimental studies have shown that acute glucose fluctuations increase oxidative stress and circulating cytokines [32]. It can be speculated that excessive oxidative stress and inflammation during acute hyperglycemia may result in decreased collateral formation. Collateral development is a multifactorial process, and all these abovementioned mechanisms may play a role in collateral formation in the course of AH.

## 5. Conclusions

In conclusion, our results suggest that blood glucose on admission may have a role in the coronary collateral development in acute MI patients. But, further studies are required to validate our results and clarify whether impaired collateral development may represent a mechanism for the increased morbidity and mortality in patients with coexisting acute MI and AH.

## 6. Study Limitations

Several limitations of the present study should be mentioned. Effects of all potential confounding factors on collateral development cannot be controlled because of the retrospective design of the study. Also, angiographically visible collaterals represent only a fraction of the total collateral vessel amount. Although the chest pain to angiography time was the same in both good collateral and poor collateral groups, complete collateral development may take several weeks. All infarct-related arteries were totally occluded, but we do not know the degree of stenosis in IRA before the index event, and severe stenosis in IRA may cause previously formed collateral vessels. Finally, this is an observational study; thus, the present study does not provide a cause-and-effect explanation for the impaired collateral development that was associated with admission hyperglycemia.

## Figures and Tables

**Table 1:** Clinical and laboratory characteristics among poor collateral and good collateral groups.

Variables	All patients (*n*=190)	Good collateral (*n*=45)	Poor collateral (*n*=145)	*p* value
Age (years)	61.64 ± 12.2	60.4 ± 13.3	61.9 ± 11.9	0.473
Men (%)	73.7	68.9	75.2	0.403
Diabetes mellitus (%)	27.4	26.7	27.6	0.904
Hypertension (%)	47.4	40.0	49.7	0.257
Any smoking history (%)	46.8	42.2	48.3	0.477
Dyslipidemia (%)	53.7	48.9	55.2	0.460
Body mass index (kg/m^2^)	21.4	21.3 ± 3.2	21.5 ± 3.9	0.177
Mean ejection fraction (%)	42.3 ± 8.6	43.2 ± 8.3	41.4 ± 9.1	0.476
Anterior MI (%)	43.7	33.3	46.9	0.036
Three-vessel disease (%)	23.2	37.8	18.6	0.026
Chest pain to angiography time (min)	282 ± 192	286 ± 205	281 ± 198	0.751
Preinfarction angina (%)	45.2	53.3	42.7	0.041
Mean admission glucose (mg/dl)	173.0 ± 80.1	148.7 ± 56.6	180.6 ± 84.9	0.008
Mean heamoglobin (g/dl)	13.8 ± 1.8	13.6 ± 1.8	13.9 ± 1.8	0.234
Mean white blood cell count	11604.8 ± 5091.3	11037.1 ± 4439.1	11780.9 ± 5279.1	0.440
Mean platelet count	244.0 ± 64.1	248.22 ± 61.65	242.80 ± 65.01	0.621
Mean total cholesterol level (mg/dl)	192.2 ± 51.1	178.0 ± 42.5	196.6 ± 52.8	0.354
Mean low-density lipoprotein level (mg/dl)	125.5 ± 33.6	117.7 ± 27.8	127.9 ± 34.4	0.046
Mean high-density lipoprotein level (mg/dl)	40.6 ± 10.0	41.0 ± 10.1	39.3 ± 9.5	0.312
Mean triglyceride level (mg/dl)	134.7 ± 103.9	120.1 ± 99.8	139.2 ± 105.0	0.718
Mean creatinine (mg/dl)	0.97 ± 0.2	0.96 ± 0.2	0.98 ± 0.2	0.692

**Table 2:** Multivariate analysis: predictors of poor collateral development in all patients.

Predictor	Odds ratio	95% confidence interval	*p* value
Admission glucose	0.994	0.989–1.000	0.049
Low-density lipoprotein	0.996	0.976–1.015	0.659
Total cholestrol	0.996	0.981–1.010	0.545
Ejection fraction	0.987	0.899–1.097	0.321
Multivessel disease	0.910	0.754–0.989	0.037
Preinfarction angina	0.932	0.816–0.985	0.043

A covariate was allowed in the model when, on univariate analysis, its *p* value was <0.25.
